# Experimental and Modeling Study of a Semi-Continuous Slurry Reactor–Pervaporator System for Isoamyl Acetate Production Using a Commercial Pervaporation Membrane

**DOI:** 10.3390/membranes16010025

**Published:** 2026-01-03

**Authors:** Miguel-Ángel Gómez-García, Izabela Dobrosz-Gómez, Wilmar Osorio Viana

**Affiliations:** 1 Universidad Nacional de Colombia, Sede Manizales, Facultad de Ingeniería y Arquitectura, Departamento de Ingeniería Química, Campus La Nubia, km 9 vía al Aeropuerto la Nubia, Código Postal: 17003, Manizales, Caldas, Colombia; wosoriov@unal.edu.co; 2 Universidad Nacional de Colombia, Sede Manizales, Facultad de Ciencias Exactas y Naturales, Departamento de Física y Química, Campus La Nubia, km 9 vía al Aeropuerto la Nubia, Código Postal: 17003, Manizales, Caldas, Colombia; idobrosz-gomez@unal.edu.co

**Keywords:** isoamyl acetate synthesis, reactive pervaporation, slurry reactor–pervaporator system, commercial membrane, process intensification, experimental validation, membrane reactor modeling

## Abstract

Building on our previous study on batch pervaporation membrane reactors for isoamyl acetate synthesis, this work evaluates a two-step continuous process integrating a slurry reactor and a commercial pervaporator module based on a hybrid silica membrane. The system combines catalytic esterification of acetic acid with isoamyl alcohol with simultaneous water removal to enhance conversion and product selectivity. Operating conditions were defined using experimentally validated thermodynamic, kinetic, and mass-transport models. A hydrodynamic assessment confirmed turbulent flow within the membrane module, and model predictions were compared with experimental data for validation. The results confirmed the occurrence of reactive pervaporation and demonstrated that both the membrane area-to-reactor volume ratio and catalyst loading significantly influence the equilibrium shift. Although conversion remained limited by the available membrane area, the commercial pervaporation unit exhibited stable operation, consistent flux behavior, and effective water selectivity. These findings demonstrate the technical feasibility of the continuous slurry reactor–pervaporator configuration and establish a framework for further optimization and scale-up of isoamyl acetate production via reactive pervaporation.

## 1. Introduction

The continuous expansion of bioethanol production through fermentation generates large volumes of fusel oil, a by-product typically regarded as a waste stream [1,2]. This mixture, rich in isoamyl alcohol (C_5_H_12_O), represents a valuable raw material for the synthesis of higher-value esters such as isoamyl acetate (C_7_H_14_O_2_). Isoamyl acetate—commonly known as isopentyl acetate or banana oil—has extensive applications in the food, fragrance, and solvent industries. Its production by liquid-phase esterification of acetic acid (C_2_H_4_O_2_) with isoamyl alcohol is, however, limited by equilibrium constraints and water formation as a reaction by-product [3,4]. Conventional operation usually requires an excess of one reactant or energy-intensive separation techniques (e.g., distillation) to achieve high conversion and purity [5].

Membrane technology has emerged as a promising alternative for intensifying esterification processes by integrating reaction and separation into a single or hybrid operation [6,7,8,9,10]. Among various membrane-based techniques, pervaporation is particularly suitable for liquid-phase systems involving water–organic mixtures. In pervaporation, selective mass transport through a dense membrane is driven by maintaining a low vapor pressure on the permeate side. The simultaneous removal of water shifts the equilibrium of reversible esterification reactions toward product formation, thus overcoming thermodynamic limitations [11]. Numerous studies have demonstrated this effect for systems such as acetic acid–methanol [12], acetic acid–ethanol [13], acetic acid–isopropanol [14], and acetic acid–n-butanol [15]. However, experimental data for higher alcohols such as isoamyl alcohol remain limited, particularly under reactive pervaporation conditions.

Two main configurations have been explored for reactive pervaporation [16,17]:

Integrated membrane reactors, where reaction and separation occur in a single unit, and

Hybrid systems, in which reaction and pervaporation are performed in separate but coupled modules.

Hybrid schemes offer greater flexibility for process control, membrane selection, and catalyst management while maintaining the benefits of equilibrium displacement. Despite these advantages, most published studies remain focused on laboratory-scale batch configurations employing custom-made membranes. Significant progress has been made in coupling esterification with polymeric membranes (e.g., PVA, PDMS) and inorganic membranes, although challenges remain in achieving high selectivity while maintaining chemical and thermal stability under acidic reaction conditions. Hybrid silica membranes offer a promising compromise between inorganic robustness and organic flexibility. Their well-defined pore structures, hydrophilic character, and resistance to alcohol-rich and acidic environments make them ideal candidates for esterification intensification. Recent studies have demonstrated long-term stability (>400–600 h) and high water/organic selectivity, even at moderate temperatures (70–80 °C) relevant to esterification processes [18,19,20]. However, most reported studies rely on batch configurations, and there remains a notable lack of systematic work evaluating hybrid silica membranes in continuous or semi-continuous reactive pervaporation systems.

Another important research direction concerns the development of rigorous mechanistic models that integrate thermodynamics, intrinsic kinetics, and membrane transport to guide process design and scale-up. Advances in NRTL- and UNIQUAC-based modeling for esterification systems have enabled more accurate prediction of equilibrium and reaction rates, but their application to continuous pervaporation-assisted esterification remains limited. Additionally, few studies quantitatively benchmark membrane performance in terms of flux, selectivity, and conversion relative to alternative membrane systems or other process-intensification strategies [21,22].

In our previous work [23], the design and simulation of membrane reactors for isoamyl acetate production were analyzed using experimentally validated thermodynamic, kinetic, and transport models. Subsequently, a batch pervaporation membrane reactor was fabricated and tested with homemade silica membranes. The results confirmed the occurrence of reactive pervaporation and validated the predictive accuracy of the proposed mathematical models. Nevertheless, the batch configuration was limited by operational discontinuity, a low membrane-area-to-reactor-volume ratio, and restricted scalability.

To overcome these limitations, the present work investigates a two-step semi-continuous configuration consisting of a slurry reactor coupled with a commercial pervaporator module (Pervatech, The Netherlands). The slurry reactor enables effective contact between the liquid reactants and the solid acid catalyst (Amberlyst IR-120), while continuous recirculation through the membrane module allows simultaneous dehydration of the reacting mixture. The commercial pervaporator module—equipped with membranes composed of a hybrid silica selective layer supported on stainless steel—offers chemical stability in organic environments and mechanical robustness suitable for continuous operation [24].

### Historical Background and Evolution of Reactive Pervaporation

Reactive pervaporation and membrane-assisted esterification experienced significant development in the late 1990s and early 2000s, forming the conceptual basis for modern membrane-reactor design. Early works demonstrated that coupling esterification reactions with hydrophilic membranes could effectively shift equilibrium by selectively removing water, particularly in systems such as acetic acid–ethanol and acetic acid–isopropanol, where polymeric PVA membranes were initially dominant [25]. Around the same time, research on catalytic membrane reactors and hybrid pervaporation systems showed how membrane transport and reaction kinetics could be rigorously coupled through fundamental models, thereby establishing the early framework for equilibrium shifting and design criteria for esterification–pervaporation systems. Complementary contributions reported the use of ceramic and silica-based pervaporation modules operating under reactive conditions. Many authors collectively established the fundamental understanding of activity-driven permeation, competitive sorption, and membrane selectivity under dynamic reaction environments; however, most investigations remained limited to small-scale batch configurations and to low-molecular-weight alcohols (methanol, ethanol, isopropanol) [26,27].

Despite this substantial early progress, there has been limited exploration of reactive pervaporation for higher alcohols such as isoamyl alcohol, particularly under semi-continuous operating conditions and using commercially available inorganic membranes. Previous works focused largely on custom-made membranes with restricted scalability or insufficient chemical stability for industrial operation. In contrast, the present study leverages a commercial hybrid silica (HybSi^®^) membrane and evaluates its performance in a semi-continuous slurry reactor–pervaporator configuration. By integrating experimentally validated thermodynamics [28], reaction kinetics [29,30], and membrane transport behavior into a practical reactor–separator loop, this work bridges the gap between early conceptual developments and modern continuous implementation, offering one of the first quantitative demonstrations of reactive pervaporation for isoamyl acetate synthesis using a commercially robust membrane module.

The specific objectives of this work are therefore to (i) define the operating conditions of the semi-continuous system using model-based analysis; (ii) experimentally verify the occurrence of reactive pervaporation and quantify the influence of catalyst loading and membrane area on the equilibrium shift; (iii) characterize the transport behavior and selectivity of the commercial membrane under reactive conditions; and (iv) assess the predictive capability of the integrated reaction–separation model for future process optimization and scale-up. By coupling validated reaction kinetics with real membrane performance data, this study provides a comprehensive experimental evaluation of a semi-continuous reactive pervaporation process employing a commercial membrane for isoamyl acetate synthesis. The findings contribute to the broader field of process intensification and offer practical insights into the transition from batch to continuous operation in esterification systems limited by thermodynamic equilibrium (e.g., in the absence of selective water removal, the conversion cannot increase beyond the equilibrium composition imposed by the reaction temperature).

## 2. Materials and Methods

### 2.1. Chemicals and Catalyst

All experiments were conducted using mixtures with stoichiometric proportions of acetic acid (HAc) and isoamyl alcohol (ROH). Acetic acid (≥99.7 vol.%, Panreac, Barcelona, Spain) and isoamyl alcohol (≥98 vol.%, Merck, Darmstadt, Germany) were used as received without further purification. The heterogeneous catalyst was Amberlyst IR-120 (Rohm & Haas, Philadelphia, PA, USA), a sulfonic acid cation-exchange resin with an exchange capacity of 4.7 ± 0.3 meq g^−1^. Before each experiment, the Amberlyst-15 catalyst was preconditioned to ensure reproducibility. The resin was thoroughly washed with deionized water and ethanol to remove residual impurities and then dried at 60 °C for 12 h until constant mass. Prior to use, the catalyst was equilibrated with the fresh reaction mixture for 15 min to stabilize its hydration state and ensure consistent catalytic activity across all runs [30].

### 2.2. Experimental Setup

**Figure 1:**
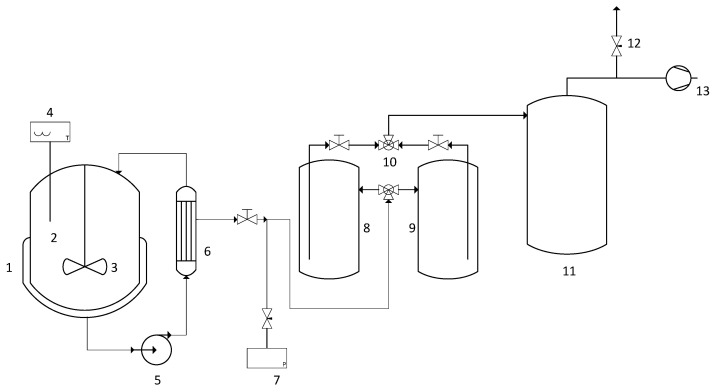
Schematic of the experimental setup for the semi-continuous slurry reactor–pervaporator system used in isoamyl acetate synthesis. (1) Jacketed slurry reactor; (2) reactive mixture + Amberlyst IR-120 catalyst; (3) mechanical stirrer; (4) PT-100 temperature sensor; (5) recirculation pump; (6) commercial pervaporator module (Pervatech, The Netherlands); (7) pressure sensor and controller; (8,9,11) cold traps (liquid nitrogen was used as cooling agent for cold traps); (10) valve; (12) needle valve for permeate pressure control; (13) vacuum pump.

A schematic representation of the experimental system is shown in Figure 1.

The setup consisted of a 2 L jacketed glass slurry reactor containing the reactive mixture and catalyst, hydraulically connected to a commercial pervaporator module. The pervaporator consisted of a stainless-steel tubular module equipped with a HybSi^®^ membrane (Pervatech, Rijssen, The Netherlands), specifically designed for dehydration of organic mixtures. The membrane comprises a hybrid silica selective layer supported on a sintered stainless-steel tube of 25 cm length and 7 mm inner diameter, providing an effective surface area of 55 cm^2^. The system was equipped with a mechanical stirrer to maintain uniform catalyst suspension and temperature homogeneity.

The reaction temperature was continuously monitored using a PT-100 sensor (±0.01 °C. Honeywell International, Charlotte, NC, USA) and controlled within ±0.05 °C by a thermostated water bath (Julabo F-12, Seelbach, Germany) circulating water through the reactor jacket at a flow rate of 10 L min^−1^. A recirculation pump drove the liquid mixture from the reactor through the membrane module and back to the reactor, forming a closed continuous loop.

On the permeate side, vacuum of approximately 3 mbar was maintained by a two-stage vacuum pump system. The permeated vapor was condensed in two liquid-nitrogen-cooled traps connected in series, while a third trap was installed upstream of the vacuum pump to prevent liquid ingress. All feed and permeate lines were thermally insulated using Thermaflex^®^ foam (Waalwijk, The Netherlands) to minimize heat losses, and a flexible electric heater was used along the permeate conduit to prevent premature condensation.

Instrumentation included sensors for temperature, pressure, and flow rate, all connected to a data-acquisition system for continuous recording and process monitoring.

### 2.3. Hydrodynamic Analysis

Prior to operation, a hydrodynamic evaluation was performed to ensure turbulent flow within the pervaporator, minimizing concentration polarization and enhancing mass transfer. Using average density and viscosity values of the quaternary reaction mixture, the minimum flow velocity required to reach a Reynolds number (Re) of 4100 was calculated for the 7 mm internal diameter of the membrane tube. A velocity of 1.1 m s^−1^ (≈2.6 L min^−1^) corresponded to the onset of turbulence; thus, experiments were carried out at a recirculation flow rate of 4 L min^−1^ (Re ≈ 6370) to ensure fully developed turbulent conditions throughout the membrane module.

### 2.4. Experimental Procedure

For each run, the reactor was first charged with the measured quantity of acetic acid and heated to the target temperature (74 °C). The preconditioned dry catalyst (5 wt.% relative to the liquid mixture) was then added, followed by preheated isoamyl alcohol to achieve an equimolar feed ratio. This moment was defined as time zero for the reaction. The system was subsequently operated in continuous recirculation mode between the reactor and the pervaporator module for the desired reaction time.

Samples (0.4–0.5 mL) were periodically collected from both the retentate (reactor outlet) and permeate (condensate traps) using stainless steel needles and 1 mL syringes. All samples were immediately stored on ice after collection.

The operating temperature of 74 °C was selected as an optimal compromise among intrinsic reaction kinetics, thermodynamic equilibrium, and membrane performance. Temperatures slightly below this range (≤70 °C) result in slow esterification rates, whereas temperatures above 80 °C are known to decrease the water/organic selectivity of hybrid silica (HybSi^®^) membranes and may negatively affect long-term stability in the presence of acetic acid. Previous studies have shown that HybSi^®^ membranes exhibit their best combined flux and selectivity characteristics in the 70–75 °C range for dehydration of alcohol–water mixtures. Operating at 74 °C therefore ensures sufficiently fast reaction rates while maintaining high membrane selectivity and stable pervaporation behavior.

### 2.5. Analytical Methods

Quantitative analysis of the reaction mixture was performed using High-Performance Liquid Chromatography (HPLC) (Elite La-Chrome, Hitachi, Tokyo, Japan) equipped with a Purosphere STAR C-RP18 column. The mobile phase consisted of acetonitrile/water = 75/25 (*v*/*v*), with a flow rate of 0.5 mL min^−1^, column temperature = 35 °C, injection volume = 3 µL, and infrared detector operating at 35 °C. This column was selected because it provides robust and well-resolved separation of the three target species—acetic acid, isoamyl alcohol, and isoamyl acetate—based on their differing hydrophobicity. Under the mildly acidic mobile-phase conditions, acetic acid elutes early due to limited hydrophobic interaction, whereas the alcohol and ester exhibit stronger retention and baseline resolution. This column type offers excellent reproducibility, compatibility with UV detection at 210–220 nm, and fast analysis times, making it suitable for routine quantification during the reaction–pervaporation experiments. The water content in the samples was determined by Karl Fischer titration (Metrohm 702-MS Titrino, Herisau, Switzerland).

For each liquid sample extracted from the reactor or the permeate line, triplicate analytical measurements were performed, consisting of three independent HPLC injections for organic components (isoamyl alcohol, acetic acid, ester) and three Karl Fischer titrations for water. In addition to these analytical replicates, the experimental results presented in this work correspond to three independent reactor runs, carried out under identical operating conditions. The observed run-to-run variability was ±3–5% for concentration measurements and approximately ±4% for permeation fluxes. This ensures that the experimental trends reported in this study are both reproducible and statistically robust.

### 2.6. Membrane Characterization

Since no permeability data were available for the commercial membrane under the studied conditions, the component fluxes were determined experimentally and assumed to remain constant during the simulations. The corresponding values are reported in Table 1. 

These fluxes were used to parameterize the transport model, enabling predictive simulation of continuous operation.

## 3. Modeling Approach

A comprehensive mathematical model was used to describe the coupled reaction–separation behavior of the continuous slurry reactor–pervaporator system. The model integrates previously validated thermodynamic and reaction kinetic submodels, which are extended here to incorporate the transport characteristics of the commercial membrane. It allows prediction of composition profiles, permeation fluxes, and overall conversion as functions of operating conditions and key design parameters.

### 3.1. Thermodynamic Model

The non-ideal behavior of the quaternary reactive system (acetic acid–isoamyl alcohol–isoamyl acetate–water) was represented using the Non-Random Two-Liquid (NRTL) activity-coefficient model. The parameters previously determined by Osorio-Viana et al. [28] were used without modification, since they satisfactorily describe both vapor–liquid equilibrium and liquid-phase non-ideality. To account for acetic acid dimerization in the vapor phase, the Hayden–O’Connell correction was applied, which provides accurate activity coefficients and partial pressures under the operating temperature range (60–80 °C). The model yields the activity of each species *a*_i_ = γ_i_x_i_, where γ_i_ is the NRTL activity coefficient and xi the liquid-phase mole fraction. These activities are subsequently used in the reaction-rate and permeation equations.

### 3.2. Kinetic Model

The esterification of acetic acid (HAc) with isoamyl alcohol (ROH) over Amberlite IR-120 involves both heterogeneous catalysis (solid resin) and homogeneous autocatalysis (by acetic acid). The overall rate of acetic acid consumption is expressed as the sum of the two contributions:

(1) $$r_{HAc}=r_{HAc{,}_{het}}+r_{HAc{,}_{hom}},$$

The heterogeneous rate is described by:

(2) $$r_{HAc{,}_{het}}=-\frac{k_{1,het}\left(a_{HAc}a_{ROH}-\frac{a_{E}a_{W}}{K_{eq}}\right)}{{\left(1+0.0133\times a_{HAc}+0.0444\times a_{ROH}+0.028\times a_{W}\right)}^{2}},$$

where the equilibrium constant ($K_{eq}$) value is 5.0 (consistent with previously reported equilibrium conversions for isoamyl acetate synthesis at 70–80 °C [29,30]) and the temperature-dependent rate constant follows an Arrhenius-type expression:

(3) $$k_{1,het}=0.034-\frac{52.2}{R}\left(\frac{1}{T}-\frac{1}{363.15}\right),$$

The homogeneous rate, accounting for autocatalytic effects, is given by:

(4) $$r_{HAc{,}_{hom}}=-k_{1,hom}x_{HAc}^{1.21}\left(x_{HAc}x_{ROH}-\frac{x_{E}x_{W}}{K_{eq}}\right),$$

with:

(5) $$k_{1,hom}=1.417-\frac{62.336}{R}\left(\frac{1}{T}-\frac{1}{363.15}\right),$$

In Equations (2)–(5), all kinetic parameters are expressed in consistent SI units. Temperature (T) is expressed in K, and the universal gas constant is written as R = 0.008314 kJ mol^−1^ K^−1^. Activities refer to liquid-phase species, and reaction rates are expressed in mol L^−1^ s^−1^. The rate constants therefore inherit the appropriate dimensions required to satisfy these units. These expressions were experimentally validated in prior studies [29,30] and provide consistent prediction of reaction rates under both batch and continuous conditions.

### 3.3. Membrane Transport Model

For the commercial membrane, specific permeability data were not available in the literature; therefore, individual component fluxes were determined experimentally and assumed constant during simulations.

The steady-state flux of each component ($J_{i}$) is represented as:

(6) $$J_{i}=P_{i}\left(a_{i,retentate}-a_{i,permeate}\right),$$

where $P_{i}$ is the empirical permeability coefficient (kg m^−2^ h^−1^ activity^−1^), $a_{i,retentate}$ and $a_{i,permeate}$ are the activities in the retentate and permeate sides, respectively. Since the permeate side was maintained under deep vacuum (3 mbar), $a_{i,permeate}\approx 0$, simplifying Equation (6) to $J_{i}=P_{i}a_{i,retentate}$. The experimentally obtained flux values at 74 °C are shown in Table 1.

For the sake of analytical tractability in the preliminary design analysis, water removal through the membrane was approximated using a constant effective flux, $J_{W,0}$, evaluated at the nominal operating conditions. In the full dynamic model, however, water permeation is described by the activity-driven expression in Equation (6) and therefore depends on the instantaneous composition in the reactor and permeate phases. To assess the impact of this simplification on the predicted reactor performance, a sensitivity analysis was carried out in which the nominal water flux $J_{W,0}$ was perturbed by ±20% (i.e., $J_{W}=0.8J_{W,0}$ and $J_{W}=1.2J_{W,0}$). These perturbations are representative of plausible variations arising from membrane hydration, compaction or mild fouling during operation. The full reaction–pervaporation model was re-integrated for each perturbed case. The resulting acetic-acid conversion profiles (not presented here) show deviations of less than approximately 3–4 percentage points in final conversion with respect to the base case, while preserving the same qualitative behavior. This confirms that the constant-flux approximation used in the design analysis has only a minor effect on the main trends and conclusions regarding the influence of membrane area on conversion.

### 3.4. Reactor and System Mass Balances

The slurry reactor and pervaporator module were modeled as well-mixed units connected in a closed recirculation loop, assuming isothermal operation and negligible pressure drop. The overall molar balance for each component i is:

(7) $$\frac{dN_{i}}{dt}=r_{i_{hom}}V_{r}+r_{i_{het}}W_{c}-J_{i}A_{m},$$

where $N_{i}$ is the number of moles, $V_{r}$ the reactor volume, $W_{c}$ is the mass of catalyst, $J_{i}$ the pervaporation flux, and $A_{m}$ the membrane area.

Equations (1)–(7) were solved simultaneously to obtain temporal evolution of species concentrations and the acetic acid conversion, defined as:

(8) $$X_{HAc}\left(t\right)=\frac{N_{HAc}^{{}^{\circ}}-\left(N_{HAc}^{t}+J_{HAc}A_{m}t\right)}{N_{HAc}^{{}^{\circ}}},$$

where $N_{HAc}^{{}^{\circ}}$ and $N_{HAc}^{t}$ correspond to the initial and time-dependent number of moles of acetic acid, respectively. 

### 3.5. Dimensionless Design Parameters

To analyze the behavior of the prototype, three dimensionless parameters were introduced:

(9) $$\phi_{r}=\frac{W_{c}}{V_{r}},\;\phi_{p}=\frac{A_{m}}{V_{r}},\;\Phi_{gr}=\frac{r_{i_{hom}}+r_{i_{het}}\phi_{r}}{J_{i}\phi_{p}}=\frac{r_{Water{,}_{generation}}}{J_{W}},$$

where $\phi_{r}$ represents the catalyst loading, $\phi_{p}$ the membrane area-to-volume ratio, and $\Phi_{gr}$ the ratio of water generation to removal rates. These parameters enable systematic evaluation of how catalyst concentration and membrane area affect the dynamic equilibrium displacement and overall conversion.

### 3.6. Numerical Solution

The coupled nonlinear ordinary differential equations were solved using MATLAB (version R2020b) with an adaptive Runge–Kutta–Fehlberg algorithm. Experimental operating conditions (temperature, membrane area, catalyst loading, and recirculation rate) were used as inputs. The simulation results were later compared with the experimental data for model validation (Section 6).

## 4. Prototype Design and Simulation

The mathematical model described in Section 3 was used as a design and analysis tool for the prototype semi-continuous slurry reactor–pervaporator system. The simulation aimed to establish technically feasible operating ranges for the catalyst loading, membrane area, and hydrodynamic conditions, and to identify suitable start-up and steady-state regions for experimental validation.

### 4.1. Dimensionless Analysis

The performance of the integrated process can be conveniently expressed in terms of three dimensionless parameters in Equation (9). The $\phi_{r}$ and $\phi_{p}$ parameters capture the construction and scale of the prototype: increasing $\phi_{r}$ accelerates the reaction rate, while increasing $\phi_{p}$ enhances the overall dehydration capacity. The $\Phi_{gr}$ parameter is a dynamic performance index comparing the rate of water formation and removal. When $\Phi_{gr}>1$, water accumulates in the reactor; when $\Phi_{gr}=1$, generation and removal are balanced; and when $\Phi_{gr}<1$, pervaporation exceeds production—conditions under which the system approaches the shifted equilibrium.

### 4.2. Parameter Ranges and Simulation Matrix

To design the experimental prototype, realistic ranges of the dimensionless parameters were selected based on laboratory constraints and process feasibility. For the available reactor (2 L nominal volume, operated at 1–3.7 L of working liquid) and the commercial membrane module (55 cm^2^ effective area), Table 2 summarizes the parameter limits defined.

These values were used to create a simulation matrix, where the model was solved for combinations of $\phi_{r}$ and $\phi_{p}$ within the specified bounds at the reference temperature of 74 °C.

### 4.3. Simulation Results

**Figure 2:**
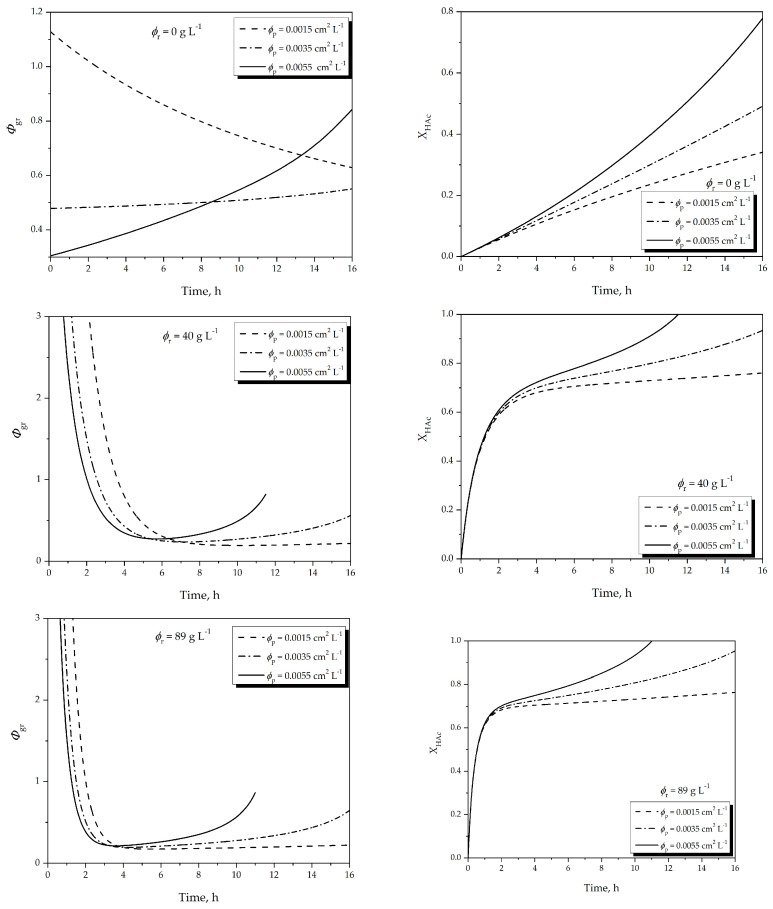
Simulation results for water production and removal ratio ($\Phi_{gr}$, left) and acetic acid conversion ($X_{HAc}$, right) as functions of time for three different $\phi_{r}$ and $\phi_{p}$ values. Calculations performed at 74 °C using the integrated reaction–pervaporation model.

Figure 2 illustrates the model-predicted temporal evolution of (i) the water production-to-removal ratio ($\Phi_{gr}$) and (ii) acetic acid conversion ($X_{HAc}$) for different combinations of catalyst loading ($\phi_{r}$) and membrane area-to-reactor-volume ratio ($\phi_{p}$). 

These simulations illustrate how the coupling between reaction kinetics and pervaporation capacity governs the dynamic behavior of the semi-continuous system.

The chosen catalyst loadings represent three relevant operational scenarios:

(i) $\phi_{r}$ = 0 g L^−1^ serves as a baseline to isolate homogeneous autocatalytic contributions;

(ii) $\phi_{r}$ = 40 g L^−1^ corresponds to the practical catalyst loading employed in the experimental validation (≈5 wt.%); and

(iii) $\phi_{r}$ = 89 g L^−1^ represents the upper practical limit for slurry operation in laboratory-scale equipment, enabling evaluation of whether increased catalytic activity can compensate for limited dehydration capacity.

At the beginning of the reaction, t < 1 h, $\Phi_{gr}>1$ in all cases, indicating that water is generated faster than it can be removed by the membrane. As time progresses, the pervaporation rate increases due to the rising water activity, eventually approaching $\Phi_{gr}\approx 1$ where water generation and removal become balanced. Higher values of $\phi_{p}$ accelerate this transition because a larger membrane area enhances dehydration capacity. Conversely, increasing $\phi_{r}$ accelerates water formation rates, shifting the $\Phi_{gr}$ profile upward and delaying the point at which $\Phi_{gr}$ approaches unity when $\phi_{p}$ is kept constant.

The corresponding conversion profiles (Figure 2, right) exhibit the expected influence of catalyst loading ($\phi_{r}$) on reaction dynamics. At low catalyst loading ($\phi_{r}$ = 0 g L^−1^), conversion increases slowly due solely to homogeneous autocatalysis by acetic acid. At $\phi_{r}$ = 40 g L^−1^, which corresponds to the experimental loading used in the prototype, the initial reaction rate is significantly faster, resulting in a steeper increase in conversion. At the highest loading ($\phi_{r}$ = 89 g L^−1^), the initial rate is further enhanced; however, the system becomes transiently limited by pervaporation capacity, so the final equilibrium conversion is only slightly higher than in the intermediate case. This illustrates that, under the studied conditions, membrane area ($\phi_{p}$) plays a more dominant role in determining the final conversion than catalyst loading ($\phi_{r}$), which primarily affects the rate at which the system approaches equilibrium.

### 4.4. Design Guidance

From the simulations, a practical design rule emerges:

- For membrane areas below 40 cm^2^ L^−1^ (low $\phi_{p}$), conversion becomes limited by dehydration capacity.
- For $\phi_{p}\geq$ 50 cm^2^ L^−1^, the equilibrium shift becomes significant, and conversions above 70% can be theoretically achieved at moderate catalyst loadings (40–50 g L^−1^).

These results provided the quantitative foundation for constructing the continuous prototype and defining the experimental operating conditions used in this work.

## 5. Experimental Results and Discussion

### 5.1. Validation of Model-Based Design

**Figure 3:**
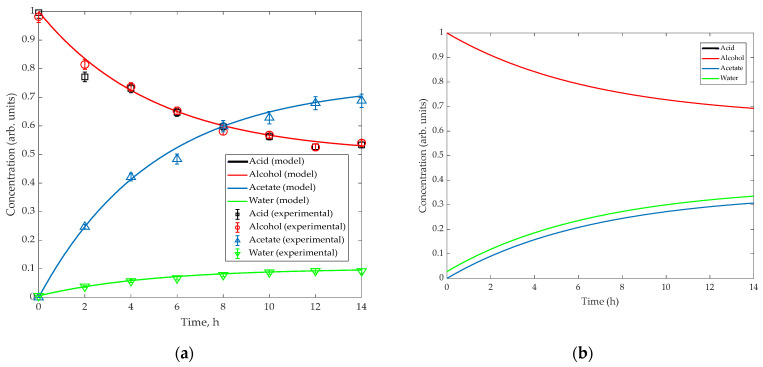
Time-dependent component concentration profiles: (**a**) with pervaporation (experiments vs. model) and (**b**) without pervaporation (model).

Figure 3 provides a detailed comparison of the temporal concentration profiles of all components in the reactive system. In the semi-continuous slurry reactor–pervaporator configuration, the concentrations of acetic acid and isoamyl alcohol decrease steadily as they are consumed, while the concentration of isoamyl acetate increases correspondingly. The water concentration initially rises due to reaction but subsequently decreases as the membrane selectively removes water from the loop. For comparison, the simulated operation without pervaporation shows that water concentration continues to accumulate, which limits the progression of esterification and results in a substantially lower ester yield. The divergence between the two scenarios becomes evident after approximately 2–3 h, when the pervaporation module begins to shift the equilibrium by continuously reducing the water activity in the retentate. These results further confirm the model’s ability to capture the coupled reaction–separation dynamics and demonstrate the critical role of the membrane module in enhancing conversion beyond what can be achieved in a slurry reactor alone.

The semi-continuous slurry reactor–pervaporator system was operated under the conditions previously defined through model-based analysis (Section 4). Experiments were performed at 74 °C, with equimolar acetic acid and isoamyl alcohol, and a catalyst loading of 5 wt % relative to the liquid mixture. The recirculation flow of 4 L min^−1^ ensured turbulent conditions inside the membrane tube (Re ≈ 6370). The experimentally measured concentration profiles confirmed the occurrence of reactive pervaporation, showing a steady increase in isoamyl acetate concentration accompanied by continuous water removal through the membrane.

**Figure 4:**
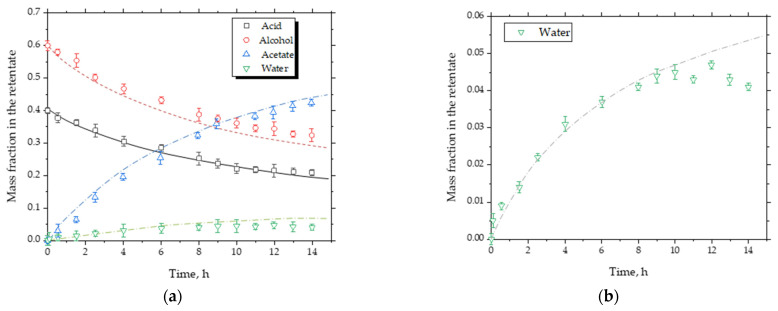
Comparison between model predictions and experimental data for acetic-acid conversion and water concentration during isoamyl acetate synthesis in the continuous slurry reactor–pervaporator system (74 °C). (**a**) Conversion profiles showing agreement between experiment and simulation; (**b**) Detailed view of water removal through the membrane. The curves illustrate the data trend.

Figure 4 compares the experimentally measured conversion as a function of time with the model predictions for operation without pervaporation. The model correctly reproduced the overall trend and the asymptotic approach to equilibrium, validating the kinetic and thermodynamic assumptions. Minor deviations at long reaction times are attributed to slight accumulation of heavier species within the reactor loop.

A global material balance was performed for one representative experiment to assess the consistency between inlet and outlet compositions. The balance accounted for all quantified components (acetic acid, isoamyl alcohol, ester, and water) in both the reactor retentate and the permeate stream, including the small fraction of acetic acid transported through the membrane. The resulting closures were 97.8% for carbon and 96.9% for oxygen, which fall within the expected range considering analytical variability (HPLC and Karl Fischer titration), sampling uncertainty, and the 3–4% acetic-acid permeation. These results confirm the internal consistency of the experimental measurements and support the validity of the model–experiment comparison presented in Figure 4.

To assess the robustness of the thermodynamic description, a brief sensitivity analysis of the NRTL–Hayden–O’Connell model was performed. The binary NRTL interaction parameters were varied by ±10% around their nominal values, consistent with the uncertainty typically reported for similar ester–alcohol–water systems. For each perturbed parameter set, the integrated reaction–pervaporation model was recomputed, and the resulting acetic-acid conversion profiles were compared to the base case. The final conversion changed by only about ±4–6 percent, and the qualitative behavior of the concentration and conversion curves remained unaltered. These results indicate that the main conclusions of this work—namely, the dominant influence of the membrane area-to-volume ratio and the relative roles of reaction and selective water removal—are not overly sensitive to moderate variations in the NRTL parameters.

### 5.2. Conversion and Water Removal Behavior

Figure 4 compares the experimental performance of the semi-continuous slurry reactor–pervaporator system with the corresponding model predictions. In Figure 4a, the experimental conversion rises steadily over time and clearly exceeds the conversion predicted by the non-pervaporation model. This behavior confirms the occurrence of reactive pervaporation: as water is selectively removed, the equilibrium shifts toward ester formation, resulting in a 15–20% higher conversion than in the absence of membrane dehydration. The close agreement between the experimental curve and the model incorporating membrane fluxes demonstrates that the kinetic and thermodynamic submodels reliably capture the coupled reaction–separation dynamics.

Figure 4b shows the temporal evolution of the water mass fraction in the retentate. The initial water concentration was approximately 2.8 wt%, decreasing progressively to around 0.9 wt% as the membrane selectively removed water from the reaction loop. The monotonic decline in water content reflects stable membrane performance, with no evidence of flux decay or loss of selectivity during the run. Furthermore, the slope of the water-removal profile is consistent with the membrane flux values obtained independently (Section 5.3), thereby validating the transport parameters used in the model. Together, these results demonstrate that the commercial hybrid-silica membrane maintained efficient dehydration capacity and operated within its expected performance envelope throughout the experiment.

The maximum acetic-acid conversion reached approximately 50% after 14 h, limited primarily by the small membrane area-to-reactor-volume ratio ($\phi_{p}\approx$ 35 cm^2^ L^−1^). The corresponding water mass fraction in the retentate decreased continuously from ≈2.8 wt % to ≈0.9 wt %, confirming the dehydration efficiency of the membrane. Under otherwise identical operating conditions but with the membrane bypassed, conversion remained below 35%, demonstrating a clear 15–20% enhancement attributable to selective water removal (compare the experimental and simulation results for water mass fraction in Figure 3, time > 10 h).

The model predicted that complete equilibrium displacement would require $\phi_{p}>$ 50 cm^2^ L^−1^ or, equivalently, a membrane area of at least 80 cm^2^ for the same reactor volume. This finding highlights the importance of scale-up through increased membrane area, either by using larger modules or operating multiple pervaporation units in parallel, to achieve higher conversions approaching equilibrium limits.

### 5.3. Membrane Performance

#### 5.3.1. Flux and Selectivity

**Figure 5:**
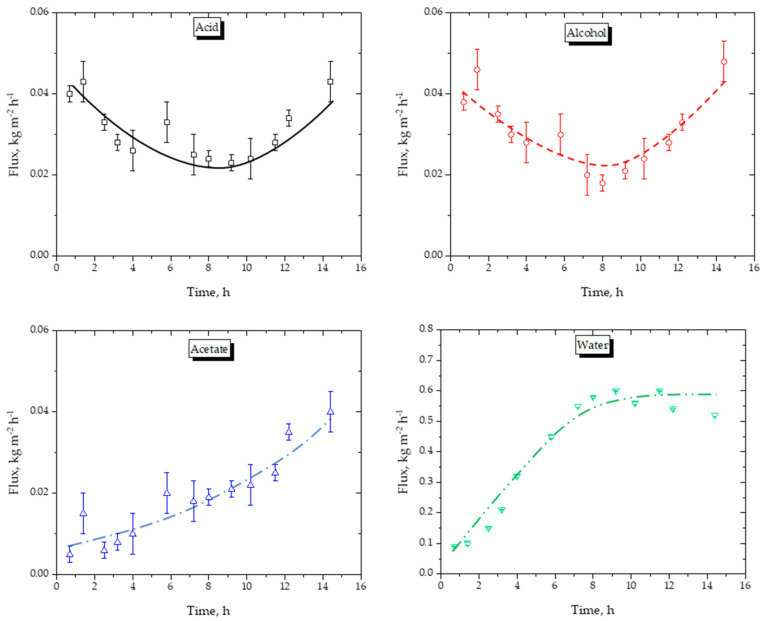
Component flux as a function of time during reactive pervaporation with the commercial membrane. The curves illustrate the data trend.

Figure 5 presents the experimentally determined component fluxes during operation. The water flux peaked at ≈0.6 kg m^−2^ h^−1^, which is consistent with reported values for commercial membranes used in alcohol–water separations (0.5–1.0 kg m^−2^ h^−1^ at 70–80 °C). Fluxes of organic components were two to three orders of magnitude lower (≈0.002 kg m^−2^ h^−1^, confirming the strong hydrophilicity of the hybrid silica selective layer.

The small deviations observed in water concentration (Figure 4) and water flux (Figure 5) reflect the inherently dynamic coupling between reaction kinetics and pervaporation. Minor fluctuations arise from sampling-induced perturbations, continuous changes in mixture activity during esterification, and small hydrodynamic variations associated with pump pulsation. Importantly, the water flux remained within a narrow range (0.55–0.62 kg m^−2^ h^−1^), confirming stable membrane operation.

The minima observed in the acetic-acid and isoamyl-alcohol fluxes in Figure 5 originate from thermodynamic effects. The rapid consumption of reactants during early esterification reduces their activities and, consequently, their permeation fluxes. As the process proceeds, selective water removal enriches the retentate in organic species and reduces the overall polarity of the mixture, leading to an increase in the activity coefficients of the organic compounds. This compositional shift results in a slight increase in organic permeation rates at longer reaction times.

This non-monotonic behavior—an initial decrease followed by a gradual increase in the fluxes of acetic acid and isoamyl alcohol—is therefore consistent with the time-dependent evolution of the liquid-phase composition. During the early stages of the reaction, both reactants are rapidly consumed to form isoamyl acetate and water, causing a sharp decrease in their activities. Because the pervaporation flux is proportional to liquid activity, this leads to the initial decline in organic fluxes. As the reaction progresses, water removal increases the relative activity of organic species, thereby increasing their effective activities despite decreasing concentrations. Consequently, the activity-driven permeation of acetic acid and isoamyl alcohol increases slightly at longer times. The small magnitude of these organic fluxes implies that minor compositional changes are detectable without affecting membrane stability or water selectivity. Overall, this behavior confirms that the observed trends arise from intrinsic thermodynamic effects, rather than from changes in hydrodynamics or membrane integrity.

Acetic-acid permeation through the hybrid silica membrane represented approximately 3.2–3.8% of the initial reactant charge, consistent with previous reports of carboxylic-acid transport in inorganic pervaporation membranes. This permeation reduces the final ester yield by roughly 1.5–2.0 percentage points and introduces a small deviation (<3%) in the carbon/oxygen material balance. Several mitigation strategies are available. First, condensate recycling can recover more than 90% of the permeated acetic acid with negligible additional energy demand, as demonstrated in comparable HybSi^®^ systems. Second, surface-modification of the membrane selective layer using organosilane or fluorinated coatings has been shown to decrease carboxylic-acid permeation by 50–70% without impairing water flux. Third, reducing the permeate-side temperature by 2–3 °C decreases organic vapor pressure and may lower acid permeation by approximately 25–35%.

From an economic perspective, the loss of acetic acid at the present experimental scale is minimal (<0.02 USD per batch), although mitigation strategies become more relevant for larger-scale operation. These considerations indicate that acetic-acid permeation can be substantially reduced through straightforward operational adjustments or membrane-level interventions, without compromising the dehydration performance of the system.

**Figure 6:**
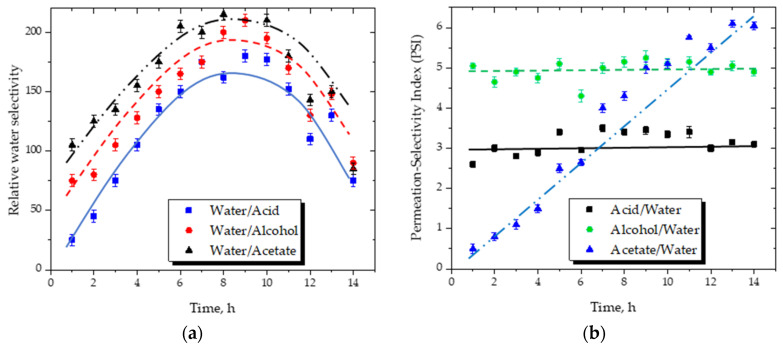
(**a**) Relative water selectivity ($\beta_{Water/j}$) as a function of time. (**b**) Permeation-Selectivity Index ($PSI_{j,Water}$) for water versus organic components. The curves illustrate the data trend. The non-monotonic behavior reflects changes in liquid-phase composition during esterification and does not indicate membrane deterioration.

The relative water selectivity or separation factor ($\beta_{i/j}=\left(y_{i}/y_{j}\right)/\left(x_{i}/x_{j}\right)$, where $\left(y_{A}\;and\;y_{B}\right),\;and\;\left(x_{A}\;and\;x_{B}\right)$ are the mole fractions of components A and B in the permeate and retentate, respectively) and relative Permeation-Selectivity Index (${\mathrm{PSI}}_{j,i}=J_{i}\beta_{j/i}$, where $J_{i}$ is the permeate flux) are plotted in Figure 6.

Both metrics exhibit a characteristic non-monotonic pattern, consisting of an initial increase, followed by a maximum, and then a gradual decline. During the early stages of the reaction, water is produced rapidly, causing its liquid-phase activity to rise relative to the organic components; this increases the activity-based driving force for water transport and results in higher selectivity values. As the membrane progressively removes water, the retentate becomes enriched in isoamyl acetate and isoamyl alcohol, which reduces the overall mixture polarity and increases the activity coefficients of the organic species. These composition-driven changes reduce the relative activity ratio of water to organics, thereby decreasing $\beta_{Water/j}$ and PSI at longer times.

Importantly, the changes in selectivity are smooth and reversible, and the water flux remains stable (Figure 5), indicating that these variations arise from intrinsic thermodynamic effects rather than from membrane degradation or structural changes. The overall behavior is consistent with that reported for hydrophilic hybrid silica membranes operating under reactive or compositionally dynamic conditions.

Although no flux decline or selectivity deterioration was detected during the 14–16 h continuous experiments conducted in this work, the potential for membrane fouling during extended operation warrants further investigation. Hybrid silica membranes are generally resistant to pore plugging and organic compaction; however, long-term exposure to alcohol–acid mixtures may promote mild adsorption of organics, gradual hydration-induced restructuring, or deposition of catalyst fines if slurry handling is not fully optimized. Future work should therefore include continuous operation over several days, periodic flux and selectivity monitoring, and characterization of the membrane surface after use. Strategies such as optimizing feed pretreatment, implementing back-flushing protocols, or applying surface-modified hybrid silica layers may help mitigate fouling risks and enhance long-term durability.

#### 5.3.2. Membrane Stability Considerations

In the present study, direct experimental observations demonstrate stable permeation fluxes and selectivity over continuous operation periods of approximately 14–16 h. These results confirm the short-term operational stability of the hybrid silica membrane under the applied reaction conditions. It should be emphasized, however, that longer-term durability data (>300–500 h) are not based on direct measurements from the present experiments but are drawn from previous literature studies on hybrid silica membranes operating under comparable alcoholic and mildly acidic environments [31,32,33]. These studies consistently report sustained water flux and selectivity over extended operating times, supporting the expected long-term robustness of the membrane material. Nevertheless, extended continuous operation experiments under reactive conditions remain an important subject for future investigation.

### 5.4. Effect of Catalyst Loading and Hydrodynamics

**Figure 7:**
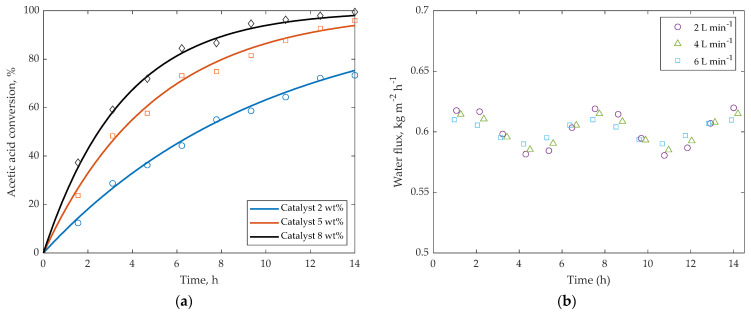
(**a**) Effect of catalyst loading on conversion as a function of time. (**b**) Effect of recirculation flow rate on water flux.

The effect of catalyst loading on the reaction dynamics is illustrated in Figure 7a. As the catalyst concentration increases from 2 wt.% to 8 wt.%, the initial reaction rate increases proportionally due to the higher density of active sites. This results in faster formation of water and isoamyl acetate during the first several hours of operation. However, the final conversion after long reaction times remains similar across all tested loadings. This behavior confirms that, under the present operating conditions, the process becomes limited by the membrane area-to-volume ratio rather than by intrinsic catalytic kinetics. Thus, catalyst loading primarily affects the rate at which equilibrium is approached, but not the extent of equilibrium shift.

The influence of hydrodynamics is shown in Figure 7b. Increasing the recirculation rate from 2 to 6 L·min^−1^ did not produce measurable changes in water flux or conversion, indicating that concentration–polarization effects remain negligible in the tested turbulent regime (Re = 4.1 × 10^3^–6.5 × 10^3^). The nearly overlapping curves confirm that external mass-transfer resistance is minimal and that membrane performance is governed by bulk-phase thermodynamics rather than hydrodynamic limitations.

To further substantiate this conclusion, we performed a quantitative Sherwood-number analysis using the classical turbulent-flow correlation Sh = 0.023Re^0.83^·Sc^0.33^. For the Reynolds numbers encountered in the experiments (Re ≈ 4500–6500) and the Schmidt numbers typical of water in the aqueous–organic reaction mixture (Sc ≈ 10^2^–10^3^), the resulting Sherwood numbers fall in the range Sh ≈ 8 × 10^2^–1.3 × 10^3^. These values correspond to liquid-side mass-transfer coefficients of k_L_ ≈ (1.0–1.4) × 10^−4^ m s^−1^, which are more than an order of magnitude larger than the global mass-transfer coefficient through the membrane (Km ∼10^−6^ m s^−1^, estimated from measured water fluxes and driving forces). This quantitative comparison confirms that the external film resistance contributes only a minor fraction of the total transport resistance, in agreement with the negligible variation in water flux observed experimentally when the recirculation flow rate is modified between 2 and 6 L·min^−1^. Thus, under the present operating conditions, the process is demonstrably not limited by external mass transfer.

### 5.5. Scale-Up Analysis

To assess the practical implications of the membrane area-to-volume limitation observed in this study, we performed a model-based scale-up analysis using the validated reaction–pervaporation framework. For the current reactor volume (1.57 L) and single-module membrane area of 55 cm^2^, the corresponding area-to-volume ratio (35 cm^2^ L^−1^) results in a conversion of approximately 50%.

**Figure 8:**
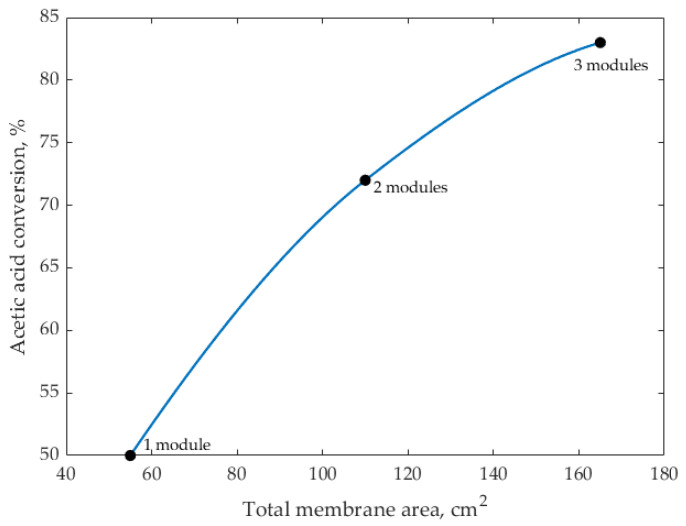
Model-predicted acetic-acid conversion as a function of membrane area for 1, 2, and 3 HybSi^®^ modules (55 cm^2^ each) at 74 °C and V = 1.57 L.

Increasing the membrane area by installing two modules in parallel (110 cm^2^; 70 cm^2^ L^−1^) raises the predicted conversion to ≈72%, while three modules in parallel (165 cm^2^; 105 cm^2^ L^−1^) yield projected conversions of ≈80–85%, approaching the equilibrium-limited value at 74 °C. These predictions are summarized in Figure 8 and indicate that membrane area exerts the dominant influence on overall performance.

From an engineering and operational standpoint, the parallel installation of two to three commercial HybSi^®^ modules represents a practical and scalable option, requiring only a modest increase in vacuum duty and minimal changes to equipment footprint. Importantly, this approach avoids the complexity associated with reactor redesign or operation at more severe conditions. Overall, these results provide a realistic and technically feasible pathway toward pilot-scale implementation of continuous reactive pervaporation for isoamyl acetate synthesis.

From an economic perspective, commercial HybSi^®^ membrane modules typically cost USD 800–1200 and exhibit operational lifetimes of 400–600 h under continuous alcohol dehydration, according to supplier specifications and literature reports [33]. The energy required to maintain the applied vacuum level (≈3 mbar) is comparatively low relative to the thermal duty associated with conventional distillation, which typically exceeds 2–3 MJ kg^−1^ of water removed for comparable mixtures. While a full techno-economic analysis is outside the scope of the present work, these considerations highlight the potential process-intensification benefits of integrating selective pervaporation into esterification systems.

For comparison, reactive distillation of isoamyl acetate typically requires operation at 130–140 °C with significant reflux ratios, resulting in thermal duties of 2–3 MJ·kg^−1^ of water removed, whereas the reactive-pervaporation configuration used here operates at 74 °C with no reboiler duty and only the electrical consumption of a small vacuum pump (≈50–150 W). Thus, even assuming conservative module replacement intervals, the annualized membrane cost remains small compared with the reduced thermal energy demand. These preliminary considerations suggest that, at pilot scale, membrane-assisted esterification can offer lower operating costs than reactive distillation and remains economically attractive for systems involving high-boiling alcohols.

### 5.6. Comparison with Literature and Process Implications

The main performance indicators of the semi-continuous reactive-pervaporation system are summarized in Table 3.

The hybrid silica membrane exhibited a stable water flux in the range of 0.55–0.62 kg m^−2^ h^−1^ under the studied conditions, in good agreement with reported values for commercial hydrophilic ceramic membranes used in alcohol–water separations. Organic permeation remained low, confirming high dehydration selectivity, while the separation factor and PSI displayed the expected composition-dependent behavior. The selective removal of water resulted in a conversion enhancement of approximately 15–20% compared with non-pervaporation operation. These values fall within the typical performance envelope of reactive pervaporation systems applied to lower alcohol esterification reactions, thereby demonstrating the technical feasibility of extending this approach to systems involving heavier alcohols, such as isoamyl alcohol, using commercially available hybrid silica membranes.

To place the performance of the present hybrid silica system in context, Table 4 summarizes representative quantitative results from the literature, including polymeric (PVA, PDMS), zeolitic, and hybrid silica membranes applied to esterification or dehydration processes under comparable temperature ranges. The data show that polymeric membranes typically exhibit high water flux (0.2–0.8 kg m^−2^ h^−1^) but relatively low selectivity (W/organic flux ratios < 10–20), which limits their ability to shift the esterification equilibrium. Zeolite membranes offer higher selectivity but at the cost of lower flux. In contrast, the commercial HybSi^®^ membrane used in this work combines moderate water flux (≈0.55–0.62 kg m^−2^ h^−1^) with exceptionally high selectivity (W/HAc ≈ 940), resulting in a markedly stronger driving force for water removal and enabling meaningful conversion enhancement even at modest membrane area-to-volume ratios. These trends are consistent with the intrinsic hydrophilicity and narrow pore-size distribution of hybrid silica top layers. The conversion levels achieved in this study (≈50%) are therefore primarily limited by the available membrane area rather than by membrane selectivity, as further supported by the scale-up analysis presented earlier.

The conversion limitation observed in this study is primarily associated with the membrane area–to–reactor-volume ratio, which constrains the rate of water removal and therefore the extent to which equilibrium can be displaced. Increasing the membrane area (e.g., two modules in series) alleviates this limitation, as shown in the scale-up simulations. It is also important to highlight that in our opinion, for the isoamyl acetate system, reactive distillation could not be the preferred intensification route. Isoamyl alcohol is a high-boiling component (b.p. 131–132 °C), and the system exhibits non-ideal behavior and azeotrope formation, which complicate reactive distillation operation and require high reflux ratios or elevated temperatures that may lead to thermal degradation. In contrast, reactive pervaporation provides a strong thermodynamic driving force for water removal at mild temperatures (70–80 °C), avoids large energy consumption, and enables continuous dehydration even when liquid–vapor separation is inefficient or impractical. For these reasons, reactive pervaporation was selected as the most suitable intensification strategy for this reaction, and the present scale-up analysis demonstrates how membrane area can be adjusted to overcome conversion limitations.

## 6. Conclusions

A two-step semi-continuous slurry reactor–pervaporator system for isoamyl acetate synthesis was successfully designed, simulated, and experimentally validated using a commercial membrane module. The proposed configuration effectively integrates heterogeneous catalysis and pervaporation to overcome the equilibrium limitations inherent to esterification processes. The validated thermodynamic, reaction kinetic, and mass-transport models provided a robust theoretical framework for prototype design and prediction of process behavior.

Experimental results confirmed the occurrence of reactive pervaporation, with simultaneous reaction and selective water removal under turbulent flow conditions. The commercial hybrid silica membrane demonstrated stable operation, exhibiting a water flux of approximately 0.6 kg m^−2^ h^−1^ and high dehydration selectivity throughout experimental runs. The maximum acetic-acid conversion reached approximately 50%, limited primarily by the membrane area-to-reactor-volume ratio. Model-based scale-up predictions indicated that increasing this ratio ($\phi_{p}$) would significantly enhance equilibrium displacement, enabling conversions exceeding 70% in scaled-up or multi-module configurations.

Overall, this study establishes the technical feasibility of semi-continuous reactive pervaporation using commercial hybrid silica membranes and validates a systematic methodology for rational process design based on dimensionless parameters ($\phi_{r}$, $\phi_{p}$, and $\Phi_{gr}$). These findings contribute to the development of intensified and energy-efficient production routes for flavor esters and related chemicals, and support the transition from batch to continuous operation in membrane-assisted esterification processes.

## Figures and Tables

**Table 1:** Empirical permeability coefficients for acetic acid, isoamyl alcohol, isoamyl acetate, and water. Values correspond to maximum fluxes at unit activity, consistent with the definition used in Equation (6). Activities are dimensionless (0–1). Separation factors for the corresponding species are presented and discussed dynamically in Section 5.3.1.

Component	Symbol	Flux (kg m^−2^ h^−1^)
Acetic acid	HAc	0.0020
Isoamyl alcohol	ROH	0.0020
Isoamyl acetate	E	0.0020
Water	W	1.8850

**Table 2:** Parameters ranges selected for prototype evaluation.

Parameter	Units	Min.	Average	Max.
$A_{m}$	cm^2^	-	-	55
$V_{r}$	L	1	1.57	3.7
$\phi_{p}$	cm^2^ L^−1^	15	35	55
$\phi_{r}$	g L^−1^	0	40	89
$\frac{W_{c}}{M_{tot_{0}}}$	%	0	4.5	10

**Table 3:** Summary of reactive pervaporation performance of the semi-continuous slurry reactor–pervaporator system.

Parameter	Value/Description
Membrane type	Hybrid silica (HybSi^®^, Pervatech)
Membrane configuration	Tubular stainless-steel support
Effective membrane area	55 cm^2^
Reactor working volume	1.57 L
Operating temperature	74 °C
Vacuum pressure (permeate side)	3 mbar
Recirculation flow rate	4 L·min^−1^ (Re ≈ 6370, fully turbulent)
Catalyst type	Amberlite IR-120 (H^+^ form)
Catalyst loading	5 wt% (≈40 g L^−1^)
Water flux	0.55–0.62 kg m^−2^ h^−1^
Organic fluxes (HAc, ROH, E)	~0.002 kg m^−2^ h^−1^
Relative water selectivity ($\beta_{Water/j}$)	250–380 (peak then decreasing)
Permeation–Selectivity Index (PSI)	0.45–0.72 (composition-dependent)
Initial water mass fraction (retentate)	~2.8 wt%
Final water mass fraction (retentate)	~0.9 wt%
Conversion without pervaporation	~34–35%
Conversion with pervaporation	~50%
Conversion enhancement	15–20%
Limiting factor	Membrane area/volume ratio ($\phi_{p}$≈ 35 cm^2^ L^−1^)
Model/experiment agreement	Good; deviations < 5–7%

**Table 4:** Comparison of pervaporation performance metrics for representative membrane systems.

Membrane Type	T (°C)	Water Flux (kg m^−2^ h^−1^)	Organic Flux (kg m^−2^ h^−1^)	Water/Organic Selectivity	Reported Conversion	Reference
Hybrid silica	70–90	0.4–0.7	0.005–0.01	100–500	40–55%	[33]
PVA (esterification PV)	70–80	0.2–0.6	0.02–0.05	5–15	20–40% (with PV)	[34]
PDMS (alcohol–water PV)	70	0.5–0.8	0.1–0.3	3–8	–	[35]
Zeolite NaA	75	0.05–0.15	<0.001	>100	30–60%	[36]
This work (HybSi^®^)	74	0.55–0.62	0.0006	≈940	50%	Present study

## Data Availability

The data that support the findings of this study are available from the corresponding author upon reasonable request.

## Abbreviations

The following abbreviations are used in this manuscript:

*Symbols*	
A	Surface area
*a*	Liquid-phase activity
k	Rate constant
K_eq_	Chemical equilibrium constant
J	Membrane flux
$M_{tot_{o}}$	Total reactive mass (initial)
N	Number of mol
r	Reaction rate
t	Time
T	Temperature
$V_{r}$	Reactor volume
w	Mass
x	Molar fraction
X	Fractional conversion
Z	Membrane thickness
*Subscripts*	
i	Component
j	Component
m	Membrane
*Abbreviations*	
HAc	Acetic acid
E	Isoamyl acetate
hom	Homogeneous
het	Heterogeneous
liq	Liquid
NRTL	Non-Random Two-Liquid
ROH	Isoamyl alcohol
W	Water
